# To Reconstruct or Not to Reconstruct: Piloting a Vietnamese and Arabic Breast Reconstruction Decision Aid in Australia

**DOI:** 10.3390/curroncol31070274

**Published:** 2024-06-28

**Authors:** Patsy S. Soon, Khouloud Kamalmaz, Verena S. Wu, Neda Karimi, Martha Gerges, Kerry A. Sherman, Afaf Girgis

**Affiliations:** 1 Psycho-Oncology Research Group, Ingham Institute for Applied Medical Research, Liverpool, NSW 2170, Australia; 2 South West Sydney Clinical Campuses, UNSW Medicine and Health, University of New South Wales, Liverpool, NSW 2160, Australia; 3 Department of Surgery, Bankstown Hospital, Bankstown, NSW 2200, Australia; 4 Institute for Communication in Healthcare, Australian National University, Acton, ACT 2601, Australia; 5 School of Psychological Sciences, Lifespan Health and Wellbeing Research Centre, Macquarie University, Macquarie Park, NSW 2109, Australia

**Keywords:** decision aid, mastectomy, breast reconstruction, culturally and linguistically diverse, Vietnamese-speaking, Arabic-speaking, qualitative, reduced inequalities

## Abstract

Currently, there are no resources to support culturally and linguistically diverse (CALD) women with breast cancer to make decisions about undergoing breast reconstruction (BR). This study evaluated the usability and acceptability of decision aids (DAs) for Vietnamese- and Arabic-speaking women. This two-phase qualitative recruited Vietnamese- (Phase 1) and Arabic-speaking (Phase 2) adult (age ≥ 18 years) women who were diagnosed with breast cancer and could read Vietnamese/Arabic. Women participated in either think-aloud telephone interviews (Phase 1) or semi-structured telephone interviews (Phase 2) and provided feedback on the DA. Interviews were audio-recorded, translated, and transcribed from Vietnamese/Arabic to English, and inductive thematic analysis was undertaken. Additionally, Arabic-speaking women completed the Preparation for Decision Making (PrepDM) scale in Round 2. Twenty-five women were recruited in two phases (Phase 1: Vietnamese-speaking women, *n* = 14; Phase 2: Arabic-speaking, *n* = 11). Three themes were developed in Phase 1: (1) DA content and reception; (2) linguistic attributes and cultural appropriateness; and (3) factors that improve the DAs’ impact. Three themes were developed in Phase 2: (1) varying perceptions of DA content; (2) linguistic and cultural suitability of information; and (3) impact of DA on decision making. Women from both phases identified areas for improvement: minimising the use of medical terminology, considering the cultural taboos associated with the word ‘breast’, and addressing remaining information gaps. Both language DAs were generally perceived as acceptable and useful in providing information about BR options and prompting women’s reflections about the suitability of BR as part of their treatment. The mean PrepDM score for Arabic-speaking women in Round 2 was 4.8/5 (SD = 0.3). Further work is needed to ensure that culturally adapted DAs take into account the myriad of information needs and health literacy levels. The key role of healthcare professionals in shared decision making among CALD populations should also be considered.

## 1. Introduction

Breast cancer (BC) is the most commonly diagnosed cancer in women, with more than 2.26 million new cases diagnosed in 2020 globally [1], and over 20,428 diagnosed in Australia [2]. Australia is culturally and linguistically diverse (CALD), with approximately 27.6% of the Australian population born overseas, and 22.8% speaking a language other than English at home of which Arabic (1.4%) and Vietnamese (1.3%) are among the most commonly spoken languages [3]. Australia’s multicultural profile bears significant implications when considering the quality of healthcare and support for diverse populations. With increased migration in recent years, this has resulted in increased numbers of migrant women diagnosed with BC, with higher percentages of women from Vietnam (21.3%) and Lebanon (19.2%) diagnosed between 2015 and 2016 compared to women from European countries [4].

Although cancer survival rates are improving [5], patients from non-English speaking backgrounds experience lower quality of life [6] and report more unmet needs compared to their English-speaking counterparts [7]. For example, an international comparative survey showed that Arab-Australian cancer survivors reported higher overall unmet supportive care needs (44.9%) compared to their Arab-Jordanian counterparts (36.1%) [8]. These disparities have significant implications when considering the quality and accessibility for cancer treatment and support in Australia.

Breast reconstruction (BR) has the potential to reduce the negative emotional and psychological impact of mastectomy and improve quality of life and body image [9]. Whilst the average BR rate in Australia has increased from 12.8% in 2010 to 29% in 2019 [10], previous research suggests that the rate is lower in South Western Sydney (9.4% between 2006 and 2015) [11], one of the most culturally diverse areas in New South Wales, Australia, in which over 68% of Vietnamese speakers and over 43% of Arabic speakers reside [3]. Common reasons for the low uptake of BR include patients not preferring to undergo BR, a lack of awareness about reconstruction as an option, patients not being offered reconstruction, and lack of access [12,13,14,15]. Evidence suggests that Caucasian women are more likely to undergo reconstruction [13], and non-English speakers were half as likely to have discussions with their healthcare team about reconstruction prior to mastectomy [16]. For Vietnamese-speaking women, one of the key barriers to undergoing BR is a lack of access to reliable information, where those who were more likely to consider BR had discussed it with their surgeon [15].

Undergoing BR entails an interactive process of decision making contingent upon opportunity (i.e., breast surgeons offering BR as an option) [17] and the information and communication preferences of the patient and her family [18]. For patients from CALD backgrounds, decision making is further compounded by language barriers, differing cultural perceptions on health, impact of religious beliefs, and involvement of family members [18]. A qualitative study conducted in Vietnam revealed that family caregivers play an integral role in decision making [19]. Family caregivers often serve as mediators between doctors and patients by taking on the responsibility of relaying information to the patient [19]. Similarly, some Arabic-Australian patients described that their family members were responsible for making treatment decisions on their behalf [18]. Whilst cross-cultural studies provide insight into some of the cultural beliefs and practices regarding decision making, it is also important to consider that these practices may not necessarily be generalisable to the entire population.

Shared decision making (SDM) therefore becomes critical as it enables decisions to be made in a collaborative way between healthcare professionals, patients, and their family members [20]. Decision aids (DAs) serve as one strategy to facilitate SDM by increasing patient knowledge of treatment options, presenting a balanced approach to the benefits and risks for treatment, enhancing preparedness for decision making, and reducing decisional conflict [21]. Trials of an online English-language DA (BRECONDA—*B*reast *RECON*struction *D*ecision *A*id [22]) developed in Australia found that women using BRECONDA benefitted by having less decisional conflict (i.e., less uncertainty in their decision making), increased knowledge, increased information satisfaction, and better preparedness for consultations with the breast surgeon, compared with women receiving general information about reconstruction [23,24]. Despite the benefits of DAs in facilitating SDM for women with BC, there is a paucity of DAs available for Australian women from Vietnamese- and Arabic-speaking backgrounds affected by cancer.

Our qualitative study focuses on Vietnamese- and Arabic-speaking women with BC, given these are the two most prevalent non-English language groups in Australia and in the South Western Sydney Local Health District (SWSLHD), where this study was undertaken. With the lack of available translated DAs, there is a dire need for adequate culturally sensitive resources to support Vietnamese- and Arabic-speaking women in their decision about whether undergoing BR is a suitable option for them. However, cultural sensitivity cannot be achieved by mere translation of resources alone. It is vital that cultural relevance is established by taking into account the norms, values, behavioural patterns, and beliefs of the target community groups to inform the design and delivery of the DA [25]. The model of cultural sensitivity [25] posits that cultural sensitivity is best achieved from consultation with the community and obtaining feedback about the content and format of the intervention. As such, DAs derived from BRECONDA were developed in consultation with Vietnamese-Australian women (Phase 1), and later refined for Arabic-Australian women (Phase 2). This paper will report the qualitative perspectives regarding the acceptability and usability of DAs adapted from BRECONDA, for Vietnamese- and Arabic-speaking women in Australia.

## 2. Materials and Methods

### 2.1. Two-Phase Qualitative Design

A two-phase study using thematic analysis [26] was undertaken to determine the acceptability and usability of a developed DA for Vietnamese-speaking women and a later translated version of the DA into Arabic, alongside an Easy English/Arabic infographic fact sheet. A community participatory approach [27] was employed, involving direct consultation with Vietnamese- and Arabic-speaking women.

### 2.2. Phase One: Vietnamese DA

#### 2.2.1. Development of the Vietnamese BR Decision Aid

The International Patient Decision Aids Standards (IPDAS) Collaboration [28] framework guided the Vietnamese DA development. The content was adapted from the existing English-language DA, ‘BRECONDA’ [22], and in consultation with stakeholders including patients with BC, clinicians, and researchers with expertise in SDM [29]. Semi-structured interviews were conducted with patients, who provided suggestions related to DA content and format [15]. Meetings were then conducted with clinicians to discuss patient suggestions and consolidate the Vietnamese DA content. Translations from English to Vietnamese were performed by NAATI-accredited translators. The adapted Vietnamese DA comprised five sections, which include exercises asking women to consider the advantages and disadvantages of each BR option and rate the importance of each statement to them (see Appendix A).

#### 2.2.2. Setting and Participants

Eligible patients were recruited from three hospitals in the SWSLHD, where Vietnamese is among the most commonly spoken non-English languages [30]. The study was approved by the SWSLHD ethics committee (HREC/17/LPOOL/24). Purposive sampling was undertaken with eligible patients who were (a) over 18 years, (b) able to read and write in Vietnamese, (c) undergoing or had undergone a mastectomy, (d) eligible to have a BR but had not yet had one, and (e) cognitively able to provide informed consent.

#### 2.2.3. Recruitment

Potential participants were identified by their treating surgeon who forwarded patients’ details to the research team for follow-up. Patients were invited by telephone by a bilingual research assistant (LDU-female). Vietnamese-speaking women who had a mastectomy in the past 10 years were also mailed study invitations, requesting that they contact the research team if they were interested in participating. Recruitment continued until all potentially eligible participants were approached. Informed consent (verbal by phone, followed up by written consent) was obtained from study participants, and they were mailed a copy of the Vietnamese DA and a demographics survey.

#### 2.2.4. Data Collection

Semi-structured telephone interviews (mean length = 37 min, SD = 18 min; range = 14–78 min) were undertaken between December 2018 and June 2019 to examine the acceptability and usability of the DA (see Appendix B for think-aloud interview schedule). Before their interview, patients were encouraged to read the entire DA, take notes, and evaluate the DA. During the interview, participants were prompted to use a think-aloud process [31] and go through each section whilst sharing their thoughts about the DA content. A bilingual research assistant (LDU) conducted the telephone interviews, at a time convenient for each patient. Interviews were audio-recorded, then translated and transcribed by a NAATI-accredited translator from Vietnamese to English.

#### 2.2.5. Data Analysis

Inductive thematic analysis (TA) was undertaken [32]. It included (1) establishing familiarity through the reading and re-reading of transcripts, (2) assigning codes to the data, (3) clustering related codes into potential themes, (4) comparing against original transcripts for reflexive recoding until data saturation, and (5) defining and naming the themes [33]. The analysis was conducted by MG, with a proportion of cases reviewed and discussed with VW as a credibility check [34]. Following completion of Phase 2 in 2023, themes from the Vietnamese-speaking group were re-examined by authors KK and VW to identify commonalities and differences with the Arabic-speaking group for subsequent comparison.

### 2.3. Phase Two: Arabic DA and English/Arabic Infographic Fact Sheet

#### 2.3.1. Development of the Arabic BR Decision Aid and English/Arabic Fact Sheet

Following Phase 1, a steering committee meeting was held with clinicians, researchers, and bilingual Arabic-speaking consumer representatives experienced in multicultural health to discuss the development of the Arabic version of the DA. Revisions were subsequently made (i.e., replacing real photographs with illustrations and the addition of a simplified infographic ‘Easy English/Arabic’ fact sheet) and sent to the steering committee for review. The revised version of the full-length DA and infographic were later translated into Arabic by NAATI-accredited translators. Both were then pilot tested with Arabic-speaking women with BC (table of contents detailed in Appendix A). The English version of the revised full-length DA and infographic were also reviewed by women who identified English as their primary language spoken at home, for which the findings will be published elsewhere.

#### 2.3.2. Setting and Participants

Following the same process as the Vietnamese-speaking group, eligible patients were recruited from the SWSLHD. The study was approved by the SWSLHD ethics committee (HREC/17/LPOOL/24). Given the recruitment challenges in Phase 1, we expanded the eligibility criteria in Phase 2 to enable further reach to Arabic-speaking women. Hence, purposive sampling was undertaken with eligible patients who were (a) aged over 18 years, (b) able to read and write in Arabic, (c) diagnosed with BC and treated with either breast-conserving surgery or a mastectomy, (d) eligible to have BR or had undergone BR, and (e) cognitively able to provide consent.

#### 2.3.3. Recruitment

The same recruitment process that was used with the Vietnamese-speaking patients was used with Arabic-speaking patients. However, several other potential interviewees were identified through connecting with representatives from other breast cancer research projects/personnel (e.g., through a multicultural health officer and the Natural Helper program). Patients were invited by text message/telephone by bilingual research assistants (NH and KK). Informed consent (verbal by phone, and written consent where possible) was obtained from all study participants. All consenting participants were mailed the participant information sheet (Arabic translation available), consent form (Arabic translation available), the detailed Arabic DA, and the English/Arabic infographic fact sheet.

#### 2.3.4. Data Collection

Semi-structured telephone interviews (mean length = 25.5 min, SD = 8.3 min; range = 13–41 min) were undertaken which examined the usability and acceptability of the detailed Arabic DA and the Easy English/Arabic infographic fact sheet, and included suggestions for further refinement and a comparison of whether the women preferred the detailed Arabic BR DA or the shorter English/Arabic infographic fact sheet (see Appendix C for interview schedule). Semi-structured interviews are beneficial in that they are not as formal as structured interviews and allow the interviewer to delve into areas relevant to the research that is driven by what the interviewee mentions, whilst keeping within the broader limit of the research topic [35]. The interviews were conducted between June 2022 and May 2023. Prior to their interview, patients were asked to read the detailed Arabic BR DA in its entirety, as well as the easy English/Arabic infographic fact sheet to be able to make evaluations. A bilingual research assistant (NH-female) conducted the first 4 telephone interviews, and another bilingual research assistant (KK-female) conducted the remaining 7 telephone interviews at a time convenient to each patient. Interviews were audio-recorded, then translated and transcribed by KK.

Patients completed the Preparation for Decision Making (PrepDM) scale which evaluates patient perceptions of how useful a DA is in preparing them for their decision making and discussing this with their doctor [36]. The 10-item PrepDM tool is rated from 1 (not useful) to 5 (very useful), with mean overall scores ranging from 1 to 5. The validated PrepDM has demonstrated excellent internal consistency (Cronbach’s alpha 0.92–0.96) [36].

#### 2.3.5. Data Analysis

Inductive TA was also undertaken for the Arabic DA interviews [32] using the same steps identified for the Vietnamese participant transcripts. Following the TA of the Arabic interviews, themes were compared against themes developed from Phase 1 (Vietnamese group) to identify commonalities and differences. The analysis was conducted by VW and KK to ensure credibility. Means and SDs were calculated for PrepDM scores in Phase 2.

## 3. Results

Of the 28 Vietnamese-speaking women approached, 5 refused participation (non-interest *n* = 4; *n* = 1 unwell), and 7 did not respond. Sixteen consented to participate, of whom 2 were lost to follow-up. In total, fourteen women completed the interview, giving an overall participation rate of 50%. The mean age of interviewees was 55.9 years (SD = 10.4), and all had undergone a mastectomy. Other participant characteristics are listed in Table 1.

Of the 22 Arabic-Australian-speaking women approached, 3 refused participation (lack of time *n* = 2, non-interest *n* = 1), 4 did not respond, and 2 were ineligible (*n* = 1 could not understand the Arabic-language content; *n* = 1 not diagnosed with cancer). Thirteen women consented to participate, of whom two subsequently withdrew due to a change of mind and other commitments. Eleven completed the interview, giving an overall participation rate of 50%. The mean age of interviewees was 52.9 years (SD = 9.6), and 7 (64%) had undergone a mastectomy at the time of the interview. The mean PrepDM was 4.8/5 (SD = 0.3). Other participant characteristics are listed in Table 1.

### 3.1. Phase 1: Vietnamese DA

Three themes were developed: (1) decision aid content and reception, (2) linguistic attributes and cultural appropriateness, and (3) factors that result in the greater impact of a DA on the target group. Indicative quotes are presented in the text and in Table 2.

### 3.2. Decision Aid Content and Reception

#### 3.2.1. Ease of Understanding

Participants reported that the DA summarised most aspects of BR in an understandable way. However, some participants noted that parts of the DA were repetitive, which caused confusion and detracted from the overall message. Conversely, one participant noted that repetition aided in understanding previously unfamiliar content.

*Because it repeats a lot. When they read for the first time, if they don’t understand, they can read the second time.*

*(V009)*

#### 3.2.2. Presentation of Medical Information and Jargon 

Given that medical terminology was used throughout the DA, some participants believed that the information was not clear, and simplification of the terminology was warranted.

*These details are very technical, like intended for professionals, not for patients.*

*(V005)*

#### 3.2.3. Addition of Supplementary Content to the DA

An important sub-theme was the need for additional supplementary content. Participants commented that financial issues were often significant factors accounted for in patients’ decisions, and therefore, the inclusion of financial information was regarded as important.

*Another important thing is the cost. After patients spend much time reading to reach the section about cost, if it is too high, they can’t do it.*

*(V015)*

Some participants felt the DA was missing practical information. They wanted suggestions they could actively implement, e.g., instructions on how to arrange an appointment with the breast and plastic surgeons if considering a mastectomy with delayed reconstruction. Two women suggested the addition of a bilingual glossary, including Vietnamese and English terms with their definition, to enable better understanding and comparison.

*So, readers can… if they don’t understand some word, they can refer to that final part. The glossary should contain the terms in both English and Vietnamese.*

*(V006)*

#### 3.2.4. Factors Related to the Readability and Display of the DA

##### Length of DA

Most participants felt that the DA was lengthy and overwhelming to read, with some participants sharing it took them over an hour to read. Reducing the number of pages and including succinct and relevant information were recommended. Several participants commented that shorter sentences would enhance overall understanding.

*I didn’t figure it out until I read it 2–3 times, but if we have to make it less wordy.*

*(V003)*

##### Format and Layout of Content

Comments on the presentation of the information were mixed, with some expressing satisfaction and others sharing that the lack of summaries made it difficult to follow.

*The content arrangement, without identifying main points, makes it very difficult for me to follow.*

*(V004)*

Suggestions were made including (1) format changes—using bolded or highlighted text to make key messages more salient—and (2) placement changes—rearranging information to other sections or combining two sections together. One participant expressed that the addition of a flowchart depicting the various reconstruction methods would better assist readers in navigating the different options.

##### Cover Page and Headings

Overall, participants were satisfied with the cover page and felt that the colours were suitable and aesthetically appealing.

*The book cover is very eye-catching and beautiful.*

*(V008)*

Several participants suggested clearer headings with either bulleted points or a numbered structure to aid in the understanding and navigation of information content.

##### Images and Figures

Participants’ views on the acceptability of photographs and figures varied. Some expressed that the images were satisfactory and realistic as they reflected their own experiences.

*These pictures look genuine. As if they’re reflecting on my case, from when the breast was still there, then cut off and left with a scar. I feel like it’s describing me.*

*(V002)*

Others felt that the current images were too negative and potentially deterred them from undergoing BR, with suggestions of more positive photos of reconstruction results.

#### 3.2.5. Overall Usefulness and Relevance of the DA

Overall, participants commented on the degree to which the usefulness and relevance of the DA could potentially impact on their decision making. Vietnamese-speaking women appreciated the opportunity to view the booklet and commented on the importance of the DA’s availability to all women in their positions.

*I find it very helpful. Those who intend to rebuild their breasts can know which option is suitable for them.*

*(V002)*

The majority of the interviewed women indicated that the DA was relevant and useful.

*Yes, it’s also good. When I got cancer, I was asked whether to do reconstruction or not. At that time, because of my age, I said “no”, and now when I read this book, I find it very interesting because I know more details about the treatment and reconstruction.*

*(V011)*

Overall, introducing a DA on BR in the Vietnamese language was acknowledged by participants to be helpful in knowing more about the BR options available to them and tailoring their decision making to their individual circumstances.

*The book analyses different methods and their effects so that we can figure out the most appropriate methods for us.*

*(V014)*

### 3.3. Linguistic Attributes and Cultural Appropriateness

#### 3.3.1. Lexical and Grammatical Issues

Participants expressed mixed feelings about the DA translation. Whilst some found that the language was easy to understand and generally consistent with what Vietnamese people would use, others said the translations were unclear, with lexical and grammatical errors. Some participants also noted the richness of the Vietnamese language and the vast range of linguistic options available.

*That’s right, our language is very rich. Depending on the level of readers’ awareness, there are some people who can understand the book, but some people have difficulties in understanding the book.*

*(V007)*

#### 3.3.2. Cultural Suitability of Words/Phrases

The DA was perceived to require more cultural sensitivity, particularly the use of more culturally acceptable terms with participants recommending using “polite” alternatives to words such as ‘breast’, which is seldom used in everyday life.

*When mentioning the word “breast” like in breast reconstruction, Vietnamese people are always reserved… “bust” and “nipple” is better.*

*(V016)*

### 3.4. Factors That May Result in a Greater Impact of the DA on the Target Group

#### 3.4.1. Timing of the Target Group Accessing the DA

Many participants expressed that early provision of the DA was vital, believing that they may have chosen another reconstruction option or made a more informed decision. Despite the variability in time since having their mastectomies, all participants believed that the DA was useful and should be distributed to women prior to undergoing a mastectomy to enable them to make an informed decision.

*Give them at the time when they go to the hospital to have a medical check-up and know that they get BC. We should give them this book so that they can read, study, and discuss with their families to make the right decision after doing the surgery.*

*(V013)*

#### 3.4.2. Supplementing the DA with Support from Healthcare Professionals

Another significant theme developed is the importance of receiving support from healthcare professionals in conjunction with receiving the DA. Two participants stressed the importance of having the DA in conjunction with advice and support from their healthcare team.

*I think it is not necessary to shorten the content in this book. If we have a support person to communicate directly, the patient will feel calm… so that they will be able to make decisions. Reading is fine, but patients will feel insecure.*

*(V007)*

#### 3.4.3. Acknowledging the Impact of Psychosocial Well-Being

Several participants noted the potential psychological strain of having BC, undergoing a mastectomy and reconstruction, and desired a specific section addressing these concerns. This would ensure that women going through this difficult decision-making period can learn more about coping mechanisms associated with BC, mastectomy, and reconstruction.

*I think if this booklet … if its purpose is to convince patients to have reconstruction, then I think the technical parts should be simpler, reduced by half, then add the psychological part, as well as more images to be more convincing, about the looks after that.*

*(V004)*

### 3.5. Phase 2: Arabic DA and English/Arabic Infographic

Three key themes were developed from the Phase 2 interviews: (1) impact of decision aid on target group’s decision-making; (2) linguistic and cultural suitability of information to target group; and (3) varying perceptions of decision aid content. Indicative quotes are presented in Table 2.

Three additional themes were also identified which did not directly address our study aims of exploring feasibility and usability but were still noteworthy when considering decision-making, and perceptions of cancer and BR among Arabic-speaking women. The additional themes are reported in the Supplementary Table S1.

### 3.6. Varying Perceptions of Decision Aid Content

#### 3.6.1. “Brevity Is the Soul of Wit”—Perceptions of the Amount of Information

There were differences in the perceptions regarding the amount of information being presented in the booklets. Some women felt both versions were complementary to one another, whilst those who were time-poor and preferred summarised versions favoured the shorter DA. Using an Arabic proverb, one of the participants noted that “brevity is the soul of wit” to highlight that there was a lot of repetition in the longer DA. Conversely, detail-oriented participants who preferred to read further into different areas of BR preferred the longer DA. Others indicated their preference for additional information such as further information about the various BC stages and how this could potentially impact on their BR options.

*The summarised one is like key points… 1,2,3… do you understand? That’s it… it stayed in my mind as well.*

*(A005)*

#### 3.6.2. Addressing Information Gaps and Pervasive Misinformation in the Community

An important sub-theme we identified is related to the usefulness of the DA in addressing information gaps and pervasive misinformation in the community. One of the interviewees mentioned that people she knew often advised her not to do BR and presented her with incorrect information about BR based on false perceptions.

*And always when they knew that I… that I want to do BR… everyone advises me… no.*

*(A011)*

#### 3.6.3. Coincides with Personal Journey

Some study participants felt that they better understood what they were going through because of the DA, and it coincided with their personal journey. Going through BC treatment alone can be isolating, and therefore having a DA in Arabic enabled the women to better understand what they were going through and the options that were available to them.

*I felt that the decision aid as I was reading it, it felt like I was reading about myself.*

*(A004)*

#### 3.6.4. Ease of Understanding of the Information Presented: Presentation and Layout of the DA

Several participants noted that the DA and infographic were easy to understand and that the presentation and layout was appropriate. However, one interviewee pointed out that improvements could be made to the presentation and layout through means such as further explanations of the pictures/illustrations. Another interviewee preferred how the pictures were presented adjacent to terms explaining them in the English/Arabic infographic.

*In general, it was all easy… and very easy to understand, and what I really liked is that they picture both the positives and the negatives in a very nice way.*

*(A001)*

#### 3.6.5. Engagement with the DA

Participants engaged with sections of the DA related to themselves more thoroughly. Whilst we asked them to read the entire DA and English/Arabic infographic, several participants noted that they did not focus on sections of the DA that were not relevant to them. This can be an issue for the usability of the DA overall, whereby some women may choose not to investigate sections of the DA that are not relevant to them.

*Me… they did not do breast mastectomy for me… and I did not focus on this… with regards to this section.*

*(A010)*

#### 3.6.6. Overall Satisfaction: Congruence with Information Received from the Healthcare Team

Satisfaction with the DA and infographic was influenced by whether the information within the DA and infographic was congruent with information received from healthcare professionals.

*It was approximately close to the explanation of the doctor… that she explained to me… no, no there is nothing.*

*(A008)*

Other women expressed overall satisfaction with the full-length DA and infographic, noting that they found them informative, and that the availability of Arabic and English translations was convenient.

*I mean, the English and the Arabic are available… I mean convenient for all people.*

*(A007)*

### 3.7. Linguistic and Cultural Suitability of Information to the Target Group

#### 3.7.1. Consideration of Cultural Taboos

The issue of cultural taboos in the DA was brought up by one of the participants who indicated that there are some personal questions being asked which might not be appropriate for people from Arabic-speaking backgrounds. Therefore, it is important to take this into consideration when DAs are developed for people of specific backgrounds.

*You are asking personal questions… that is very embarrassing. So, if anyone is going to hear or read about… this information… they will take a first impression… that there is something wrong.*

*(A011)*

#### 3.7.2. Translation Issues

Another issue discussed in some of the interviews is regarding the terms being used in the DA, with some participants noting that they were unsure what specific terms meant. For example, some women found the term “bags”, which was referred to in the DA section on implants, difficult to understand.

*When they implant… they want to put implants inside and they put in it… something like a gel or water or silicone… I do not know exactly why they have used the term “bag”.*

*(A003)*

#### 3.7.3. Importance of Translators in Facilitating Discussions

One of the issues that the research team noticed when conducting the interviews was that patients often had doctors who did not speak Arabic. This meant that they relied heavily on translators communicating between patients and doctors. Having a DA in Arabic can be especially useful in such circumstances, whereby the majority of the information related to BR options can be available upfront.

*I: And your doctor, did she speak Arabic?*

*P: No.*

*(A005)*

#### 3.7.4. Increasing Accessibility to the Wider Community

Participants also noted that it would be beneficial to increase the accessibility of the DA and infographic to reach the wider Arabic-speaking community. Whilst participants noted the importance of having these resources accessible online, they also suggested that the DA be available through different means, such as in hospitals, if cost permits. One participant indicated that some women may not know how to use technology appropriately or may have internet access issues; therefore, both hard copies and soft copies are needed.

*So, I advise that… they… do copies and keep them in the hospital… and the doctor to explain… and… this booklet… as well… it gives more information.*

*(A009)*

#### 3.7.5. Translation Accuracy Facilitates the Effective Communication of Information

Many of the participants we interviewed indicated that they were happy with the translation accuracy in the DA and the information sheet and felt that this made it easier for them to get the overall message of what the DA is trying to convey.

*I understood… I read and understood… I took the synopsis… the answer or the outcome was reached.*

*(A005)*

#### 3.7.6. Varied Levels of Understanding despite Translation Accuracy

There were varied levels of understanding of the DA, despite translation accuracy. The reason behind this is that some of the women interviewed felt that specific medical jargon was difficult to understand. This was influenced by the type of medical jargon being used (i.e., less common terms were more difficult to understand) and the educational/professional backgrounds of the woman being interviewed.

*Oh, there was the written information that had medical jargon. This part, of course I did not know about clearly.*

*(A002)*

### 3.8. Impact of the Decision Aid on the Target Group’s Decision Making

#### 3.8.1. Balanced Understanding of Reconstruction through Exposure to the Advantages and Disadvantages of Each Option

Study participants felt that the DA improved their knowledge and gave them a balanced understanding of what reconstruction is. The inclusion of both the advantages and the disadvantages of different BR options was noted by several participants to be the reason.

*What I really liked is that they picture both the positives and the negatives in a very nice way.*

*(A001)*

#### 3.8.2. Facilitating Decision Making

Study participants also felt that the DA facilitated their decision making by helping them to understand more about BR.

*And this is the one that gave me useful information… regarding taking the decision… I was hesitant about… the decision about… about the surgery.*

*(A007)*

#### 3.8.3. More Confidence in Consulting with the Doctor and Seeking Help

One interesting theme that we found was related to how the DA gave the participants more confidence in consulting with their doctor. One participant stressed the importance of seeking help and not being fearful, because early intervention can be better for BC treatment.

*And to encourage the women to not be fearful, that you need to go to the doctor, you need to check up on yourself. Do not delay yourself and say “no, later”. And to say that “it is okay this is nothing”.*

*(A004)*

#### 3.8.4. Unaddressed Knowledge Gaps about Breast Reconstruction

Although the project participants did note that the DA was extremely helpful overall, some highlighted several unaddressed knowledge gaps that the DA had. Specific areas that participants had difficulty in terms of recalling included “what flap reconstruction means”, and one participant specifically noted that she would have liked more information about how breast size impacts BR options.

*It didn’t… they didn’t explain with regards to different conditions… with regard to sizes of the initial breast… the much larger one… the smaller one… the middle one.*

*(A006)*

#### 3.8.5. Prompted Reconsideration of Their Decision

Some participants found the DA and the English/Arabic factsheet useful in that they prompted reconsideration of their BR-related decisions.

This was not the case for all the women, and one woman noted that upon reading about the disadvantages of different BR options, she no longer wanted to bring up the topic with her doctor. Nevertheless, the content of the DA was useful in that it did prompt several participants to reconsider if they were or were not going to undertake BR.

*The matter has finished… I have a check-up now in the 12th month [December]… this year… I will bring up the topic and see… if there is a possibility… I mean that some can start with the surgery… then why not?*

*(A005)*

#### 3.8.6. Offers Psychosocial Support/Reassurance about Available Options

Amongst the noted benefits of the DA is that it offered participants psychosocial support and reassurance about the various alternative options that are available for them. One participant highlighted that being diagnosed with BC and going through treatment can increase the chances of developing depression. By having a DA that simplifies complex information and offers psychosocial support to women going through this difficult period, women can feel a sense of reassurance about their decision making.

*Especially when a person gets this illness… because there is a little bit of depression… and a person becomes… they simplify the matter… honestly.*

*(A010)*

## 4. Discussion

A two-phase qualitative study was undertaken to examine the feasibility and acceptability of a Vietnamese-language (Phase 1) and Arabic-language (Phase 2) BR decision aid for women in Australia. Fourteen Vietnamese-speaking women and 11 Arabic-speaking women took part in semi-structured interviews and provided their feedback. Overall, the DA was perceived as useful by Vietnamese- and Arabic-speaking women in enhancing their awareness of available BR options. However, issues pertaining to acceptability and usability were raised, which are discussed in further detail below.

Both DAs were mostly perceived as useful in providing information about various BR methods and prompting reflection on whether BR was a suitable option. However, women in both groups identified remaining knowledge gaps that were unaddressed, and our findings suggest some cross-cultural differences in unmet information needs. Knowledge gaps identified by Vietnamese-speaking women in our study included information about psychological, financial, and practical support (e.g., knowing how to arrange an appointment with the breast surgeon), whereas Arabic-speaking women desired more information about the recovery from BR and the disease itself, and how their individual circumstances (e.g., breast size) affect their BR options. Previous research involving Vietnamese women undergoing chemotherapy, however, suggested that they also need information on whether the cancer has returned, the meaning of their test results, as well as the treatment side-effects they should report to their healthcare team [36]. The unmet information needs expressed by the Arabic-speaking women in our study reflect previous findings that Arabic-speaking patients commonly report unmet information needs pertaining to cancer generally and treatment effectiveness and side-effects [37]. These findings illustrate that informed decision making for CALD patients is contingent upon having sufficient and comprehensible information about coping with the physical and emotional consequences of treatment [38]. The need for further psychological support was acknowledged and addressed in the original web-based BRECONDA where this included sections about stress recognition and management [39]. The remaining knowledge gaps identified in our study warrant awareness from healthcare professionals that they may need to provide CALD patients with further options for support [38]. Future revisions to the DAs could also include stress recognition and management strategies derived from BRECONDA [39].

Another shortfall identified by both language groups in our study was that the medical jargon contained in the DAs (despite their translation) marred their ability to understand the DA content. Arab-Australians have previously reported their preference for information about treatment to be communicated without technical explanations [8]. This was a sentiment echoed by both Vietnamese- and Arabic-speaking women regarding the full-length DA. We considered the feedback from Phase 1 and subsequently developed an Easy English/Arabic infographic in addition to the full-length DA and piloted this among Arabic-speaking women in Phase 2. The infographic was seen as acceptable by Arabic-speaking women; however, women expressed mixed preferences for each version. These findings highlight the diversity in information preferences and the importance of catering to literacy disparities when engaging in SDM. Health literacy is therefore a critical factor to consider in the context of developing DAs for CALD communities, where this encompasses an individual’s ability to access health resources and use them effectively [40]. DAs that are not comprehensible would have limited utility among CALD individuals with limited health literacy. Hence, it is integral to accommodate for varying literacy levels and utilise strategies (such as the infographic we developed) to enable the clear communication of treatment options.

Notably, the findings illuminated the importance of women having support from their healthcare professionals, as this aided in their decision making. Vietnamese-speaking women shared that receiving the DA in conjunction with advice from their healthcare team would provide them with additional reassurance when making their decision. Arabic-speaking women, on the other hand, felt more confident consulting with their doctor after consulting the DA, which was reflected in the overall high mean PrepDM score after use of the DA. Our findings indicate that an SDM approach may benefit immigrant groups, especially when patients are supported by their clinicians whilst considering treatment options [41]. Systematic review findings indicate patients who experience disadvantage and low literacy benefited more from SDM interventions compared to those who had a higher literacy, education, and socioeconomic status [42]. Therefore, the impact of DAs on informed decision making may be enhanced with a more collaborative approach between patients and clinicians.

### 4.1. Strengths and Limitations

A key strength of our study is that we adopted an iterative approach by consulting with key stakeholders (which included patients, researchers, healthcare professionals, and community members working in multicultural health) to guide the early development of the DAs. Stakeholders provided initial guidance on the content and layout of the first iteration of the DA. Following this, our two-phase qualitative approach enabled us to seek feedback directly from Vietnamese- and Arabic-speaking women to inform the future revisions needed. However, there were also limitations to this study. The static paper-based DA presented to the participants in the present study lacked the interactive components of the original online BRECONDA where participants could tailor the quantity of information by requesting more detailed information via clicking [39]. Conversely, the DAs in our study meant that all participants received the same quantity of information, which may explain the variation in the preferred amount of information.

The population recruited was also limited to hospital settings within the SWSLHD in Australia and represented a small cohort of the Vietnamese- and Arabic-speaking communities. Hence, views are not representative of Vietnamese- and Arabic-speaking populations globally, and the transferability of the findings should be interpreted with caution. The think-aloud interviewing process was also used in Phase 1 where Vietnamese-speaking women were asked to review each section in turn and share their comments with the interviewer as they reviewed the DA. This may have also resulted in significant variability in the length of the interviews for the Vietnamese group (ranging between 14 and 78 min) as some women may have needed more time to process the DA content and provide their feedback.

To overcome the potential limitations of the think-aloud process, we adopted a semi-structured interview approach in Phase 2 as this was deemed more suitable for Arabic-speaking women and sought their feedback about specific facets of the DA we wanted to interrogate (i.e., level of understanding, usability, format, language, and comparison with the Easy Arabic booklet). We still noted the variability in the length of the interviews (range 13–41 min) in Phase 2, which may be attributed to individual preferences for the amount of feedback they wished to contribute. Furthermore, the use of the two interviewing processes may have elicited different types of feedback, thus limiting an adequate comparability of the findings. We also added the PrepDM scale in Phase 2 to better understand the impact of the DA on patients’ perceptions of the DA in preparedness for decision making and discussion with their doctor. However, the results should be interpreted with caution as this was not assessed among Vietnamese-speaking women in Phase 1, which limits comparability. Future research is needed among a larger and diverse cohort to better understand the impact of the DAs on decision preparedness.

### 4.2. Practical Implications

Our findings illustrate the importance of healthcare professionals’ roles in SDM which is characterised by (1) patient participation, (2) exploring and comparing treatment options, (3) assessing patient values and preferences, (4) reaching a decision with the patient, and (5) evaluating the patient’s decision [43]. DAs can be useful in providing information on the advantages and disadvantages of each treatment option and prompt patients to consider their values and preferences [44]. However, the role of the clinician is critical to SDM, particularly when working with CALD communities, where adequate care goes beyond mitigating language barriers [45].

Although interpreters can assist with the communication about treatment options, a more holistic approach is needed, including the consideration of social and contextual factors such as whether patients come from a collectivistic culture and prefer for their family members to be involved in the discussion [46]. Healthcare professionals can benefit from training in cultural competence characterised by self-assessment, learning, and awareness of the cultural nuances that underpin health decisions [47]. Additionally, bilingual patient navigators can assist healthcare clinicians with providing CALD patients and families the additional support they need [48].

Our findings suggest that further modifications to the Vietnamese- and Arabic-language decision aids are required to enhance usability and acceptability, and it is unlikely that the effects on decision making will be realised unless this is addressed first. One potential strategy is to complement the paper-based DA with interactive tools such as videos, to cater to the diverse information preferences expressed by the women in this study. Moreover, support groups can serve as another option for women to discuss their treatment options with peers and provide the additional reassurance they need in their decision making.

Lastly, previous trials of BRECONDA have indicated efficacy in reducing decisional regret among women with breast cancer [23,24]. Therefore, further research is needed to explore the immediate and long-term impact of the Vietnamese- and Arabic-language DAs on patient knowledge and decisional satisfaction.

## 5. Conclusions

Our study reported the qualitative perspectives related to the acceptability and usability of DAs for Vietnamese-speaking and Arabic-speaking women in Australia. Our study highlights that culturally adapted DAs are useful for disseminating knowledge about treatment options for people from CALD backgrounds, especially in multicultural contexts like Australia. Despite the limitations of our study and the suggestions for further improvements made by some of the participants regarding the piloted DAs, it remains beneficial as it opens the pathways for DAs to be developed in other health-related contexts. Our study also provides suggestions about factors (positive aspects and aspects worth improving) that can impact the acceptability and usability of DAs for women of CALD backgrounds. We therefore encourage researchers to consider the development of culturally appropriate DAs for (1) other geographic contexts, (2) other health-related contexts, and (3) to extend DA development beyond women as target groups.

## Figures and Tables

**Table 1:** Participant characteristics.

Demographic Characteristics		Vietnamese N = 14 *n* (%)	Arabic N = 11 *n* (%)
**Age (median, range)**		54.5 (42–74)	50.5 (35–73)
**Country of Birth**	Australia	-	1 (9)
	Vietnam	14 (100)	NA
	Iraq	NA	6 (55)
	Lebanon	NA	3 (27)
	Egypt	NA	1 (9)
**Relationship Status**	Single/Separated/Widowed	4 (29)	4 (36)
	Married/Partnered	10 (71)	7 (64)
**Education ^a^**	Primary	3 (21)	2 (18)
	Secondary	7 (50)	2 (18)
	Tertiary	4 (29)	4 (36)
	Missing data ^b^	NA	3 (27)
**Employment Status ^a^**	Employed	7 (50)	2 (18)
	Retired	6 (43)	2 (18)
	Other	1 (7)	4 (36)
	Missing data ^b^	NA	3 (27)
**Primary Language Spoken**	Vietnamese	14 (100)	NA
	Arabic	NA	11 (100)

NA—Not Applicable; ^a^ Details about education and employment status from the Arabic-speaking patients were confirmed after the interviews through follow-up text messaging. Hence, their status may have changed since the time of the interviews. ^b^ Missing data attributed to non-responses during follow-up.

**Table 2:** Themes, subthemes, and illustrative quotes from Phases 1 and 2.

Phase 1: Vietnamese-Speaking Women (N = 14)	Phase 2: Arabic-Speaking Women (N = 11)
**1. Decision aid content and reception** ***1.1. Ease of understanding*** *These parts are repetitive… I mean, all the parts 1, 2, 3, 4, 5 sometimes overlap each other.* (V005) *Yes. It’s easy to understand. I can know more about the advantages and disadvantages*. (V009) ***1.2. Presentation of medical information and jargon*** *The next part is part 2, about breast reconstruction using implants… It’s messy… not clear, I don’t know what they want to say.* (V001) *But I think this seems inaccurate. It says … for example, when I had an expander inserted, I asked if it had an expiration date, because the expander has a time limit, then there must be a time limit for delay reconstruction.* (V004) ***1.3. Addition of supplementary content to the DA*** *You have to consider the money thing. For example, if the patient doesn’t have such difficulties, then … surely she will have reconstruction sooner or later, but she does not know that … how much the government would support her and how much she would spend on reconstruction.* (V004) *It should mention patient psychology, making patients feel reassured that the disease doesn’t affect their lives much.* (V005) ***1.4. Factors related to readability and display of the DA*** Length *Make it briefer so that the patients can make a decision easily and see things gently. This one is confusing, making the patients hesitate and wonder whether to do reconstruction or not.* (V011) Format and layout of content *We should highlight this section, or we can make it involved and add the note below. After that, we have to include that sentence in this note… you can highlight or bold so that readers will know it’s important.* (V001) *I would like to learn about the methods carefully. I don’t think the contents are either too short or too long. Patients like us really want to read many materials like this one. The repetitive sections should be replaced by more useful information.* (V014) Cover page and headings *It’s good, as pink is also used for the ribbon representing breast cancer.* (V003) *This booklet should have something like number format I, II… 1, 2… to make it easier for readers to follow*. (V004) Images and figures *There should be another figure showing how the breast looks after being successfully reconstructed again. That will interest patients. What is shown here is too scary.* (V004) *No problem. It’s good that there’s an illustration like this for patients to imagine how it will be … like this part … so they know if they choose this way, how they will look, helping them decide whether or not they have reconstruction.* (V005) ***1.5. Overall usefulness and relevance of DA*** *Yes, it’s also good. When I got cancer, I was asked whether to do reconstruction or not. At that time, because of my age, I said “no”, and now when I read this book, I find it very interesting because I know more details about the treatment and reconstruction. It’s very good.* (V011) *Well, I think this book helps patients like us to consider different reconstruction methods. There are suitable treatments for each patient’s situations.* (V014)	**1. Varying perceptions of decision aid content** ***1.1. “Brevity is the soul of wit”: perceptions of the amount of information*** *As they say in Arabic “brevity is the soul of wit”.* (A001) *For example it be summarised about stage 0 stage 1 stage 2. Maybe if they explain about these things… the woman… it encourages her to do the mammogram.* (A009) ***1.2. Addressing information gaps and pervasive misinformation in the community*** *There is a lot of incorrect information that is spread in the Arabic community.* (A011) ***1.3. Coincides with personal journey*** *I felt that I felt that the decision aid as I was reading it, it felt like I was reading about myself.* (A004) *What I felt for example, that is what happened to me, what is the emotion/feeling. It made me understand, that is, why I was in pain.* (A008) ***1.4. Ease of understanding of the information presented: presentation and layout of the DA*** *In general, it was all easy and very easy to understand, and what I really liked is that they picture both the positives and the negatives in a very nice way.* (A001) *It clarifies the types of breast reconstruction, but if only you had further explanation as well. What we need is more illustrative pictures after reconstruction. How will it look?* (A006) ***1.5. Engagement with the DA*** *I just read what is directly related to me. I did not read the rest of the booklet*. (A002) *So now, when I read the booklet, honestly when I read it… that is very briefly just so that I can take some information*. (A007) ***1.6. Overall satisfaction: congruence with information received from the healthcare team*** *It was approximately close to the explanation of the doctor… that she explained to me… no, no there is nothing.* (A008) *I mean the booklet is very nice, but there are things that it is repeating the same.* (A009)
**2. Linguistic attributes and cultural appropriateness** ***2.1. Lexical and grammatical issues*** *But on the other hand, this book doesn’t use Vietnamese grammatical structure, so it’s hard for me to read it. I think when a person get sick, they’re very distressed, so I don’t know … if I had read it when I were sick like that, I don’t know if I can understand it.* (V004) *Firstly,”tái tạo ngực” sounds like … more Vietnamese. And the word “vú” shouldn’t be used for written language, but more spoken language. As for a book, “ngực” would be better. Because in this context, when you say “ngực”, people would know it’s breast.* (V005) ***2.2. Cultural suitability of words/phrases*** *The word “bust” in Vietnamese is often used informally. In the book, we should use a more polite word, which is more suitable with writing style than the word “breast”. When we hold the book and read it, we’ll see it fits the Vietnamese writing style.* (V011)	**2. Linguistic and cultural suitability of information to the target group** ***2.1. Consideration of cultural taboos*** *Ahh…one that caught my mind… you are asking personal questions that is very embarrassing. So, if anyone is going to hear or read about this information they will take a first impression that there is something wrong.* (A011) ***2.2. Translation issues*** *They want to put implants inside and they put in it something like a gel or water or silicone. I do not know exactly why they have used the term “bag”.* (A003) ***2.3. Importance of translators in facilitating discussions*** *There was a translator… there was no Arabic.* (A005) ***2.4. Increasing accessibility to the wider community*** *So, I advise that they do copies and keep them in the hospital and the doctor to explain… and for example this booklet as well it gives more information.* (A009) *They maybe can try to see I take a lot of information from YouTuber or for example from NSW Government Health.* (A011) ***2.5. Translation accuracy facilitates the effective communication of information*** *There are people that are like us and there are people that are even better. Regarding me, I am a home maker and I do not have this scientific culture. I haven’t for example… I ascended in one step… but in regard to my life… I mean it is normal. (A005)* *The word itself for some people will find it a bit difficult for them.* (A007) ***2.6. Varied levels of understanding despite translation accuracy*** *Oh, there was the written information that had medical jargon. This part, of course I did not know about clearly. So, when they speak about hormones and things like that… these stuff, I do not know them.* (A002) *In my experience, I didn’t find it difficult. It depends on each person, them and their… maybe their studies or their experience. I don’t know, I didn’t find it difficult.* (A003)
**3. Factors that may result in a greater impact of the DA on the target group** ***3.1. Timing of the target group accessing the DA*** *If I could turn back to the time before my surgery and had a chance to read this book, it would help me know more, and I would have a chance to choose a better new surgery method.* (V009) *Give them at the time when they go to the hospital to have a medical check-up and know that they get breast cancer. We should give them this book so that they can read, study, and discuss with their families to make the right decision after doing the surgery.* (V013) ***3.2. Supplementing the DA with support from healthcare professionals*** *That’s right. It is so painful and sad to have a body part removed. No one is happy in that situation. Still, consider, but encourage. The specialist needs to give comments. Suggestion and encourage the patient to be brave.* (V013) *I found this document suitable for the patients to read just before the discussion with the doctor about the operation*. (V015) ***3.3. Acknowledging the impact of psychosocial well-being*** *I think if this booklet… if its purpose is to convince patients to have reconstruction, then I think the technical parts should be simpler, reduced by half, then add the psychological part, as well as more images to be more convincing, about the looks after that.* (V004)	**3. Impact of the decision aid on the target group’s decision making** ***3.1. Balanced understanding of reconstruction through exposure to the advantages and disadvantages of each option*** *What I really liked is that they picture both the positives and the negatives in a very nice way*. (A001) *The difference between them and what are the pros and cons.* (A007) ***3.2. Facilitating decision making*** *Telling you was very useful.* (A001) *No. It was good… the person, he/she benefits from it and benefits others. I mean, in the things that the person has experience in.* (A004) ***3.3. More confidence in consulting with the doctor and seeking help*** *I: Okay. Does it prepare you to talk to your doctor about what matters most to you?* *P: Yes. I get comfortable. Correct.* (A004) ***3.4. Unaddressed knowledge gaps about breast reconstruction*** *They didn’t explain with regards to different conditions, with regard to sizes of the initial breast, the much larger one, the smaller one, the middle one.* (A006) ***3.5. Prompted reconsideration of their decision*** *No, that is it. I mean the matter has finished. I have a check-up now in the 12th month this year. I will bring up the topic and see… if there is a possibility, I mean that some can start with the surgery, then why not?* (A005) ***3.6. Offers psychosocial support/reassurance about available options*** *Especially when a person gets this illness because there is a little of depression and a person becomes… I mean, they simplify the matter. (A010)* *Social and that… it is very nice when a person is ill and how they encourage a sick person so that to do the surgery and to benefit from it through….* (A009)

## Data Availability

The authors confirm that the data supporting the findings of this study are available within the article and its Supplementary Materials.

## Supplementary Materials

The following supporting information can be downloaded at https://www.mdpi.com/article/10.3390/curroncol31070274/s1, Table S1: Additional themes identified in Phase 2 (Arabic-speaking women), Figure S1: Examples of decision aid content (English), Figure S2: Examples of decision aid content (Vietnamese), Figure S3: Examples of decision aid content (Arabic), Figure S4: Easy English/Arabic infographic content.

## Appendix A. Breast Reconstruction After Mastectomy Decision Aid Information Content

**Section**	**Content and Description**
**Introduction**	*Breast reconstruction after mastectomy* Opening statement about the purpose of the decision aid booklet, reassurance about how there are no right or wrong decisions, and statement that this should not replace health advice and that decisions should be made in consultation with their surgeon. Includes an illustrative quote from a 61-year-old Vietnamese woman about the challenges of language barriers in receiving information she needs.
**Section 1**	*(a) What is a mastectomy?* Brief description of what a mastectomy is. Includes photograph of a breast after a mastectomy. *(b) What is breast reconstruction?* Brief description of what breast reconstruction is and its purpose. Includes photographs of breast prostheses and the insertion of a prosthesis inside a bra. *(c) Who can undergo breast reconstruction?* Brief description of who might be considering breast reconstruction including someone who has been recently diagnosed and about to have a mastectomy, or someone who had been diagnosed some time ago and already had their mastectomy. *(d) Can I choose not to have breast reconstruction?* Brief description that some women choose to use external breast prostheses as opposed to having breast reconstruction. *(e) Thinking about breast reconstruction options* Includes a brief overview of the timing for breast reconstruction (immediate and delayed), types of breast reconstruction (implants, flap reconstruction), and nipple/areola reconstruction. Includes photographs of patients who have decided on different options (e.g., left breast removed without skin and using immediate reconstruction) and diagrams of skin and mechanisms of the latissimus dorsi flap reconstruction procedure, transverse rectus abdominal muscle (TRAM) procedure, and deep inferior epigastric perforator (DIEP) flap procedure.
**Section 2**	*(a) Immediate reconstruction* This section included information on all the different types of immediate reconstruction options including a skin/nipple-sparing mastectomy with a one-stage implant-based reconstruction, flap reconstruction with latissimus dorsi, or DIEP flap construction. Photographs of patients with immediate reconstruction, and TRAM procedures are included. *(b) Delayed reconstruction* This section included information on the different types of delayed reconstruction options including the two-stage implant-based reconstruction and flap reconstruction with either latissimus dorsi, TRAM, or DIEP. Includes diagram of the breast implant procedure. *(c) Summary* This section provided a summary of the factors that the doctor will consider when advising on the type and timing of breast reconstruction. *(d) Things to consider about the timing of breast reconstruction* This section provided a summary of considerations for the timing of breast reconstruction, such as the type of treatment the patient is undergoing (radiotherapy or chemotherapy), timing of the treatment, and doctor’s advice.
**Section 3** **Possible complications**	*(a) What might go wrong with implants?* Brief opening statement about how there may be a low risk of getting an infection in the reconstructed breast area, along with other complications. *(b) What might go wrong with implants? Capsular contracture* Description of capsular contracture, i.e., tightening of the implant and distortion of the breast shape as normal scar tissue forms around the implant. Photograph of a patient with differences in breast size after breast reconstruction and adjuvant radiotherapy. *(c) Other minor complications with implants* Description of other minor complications that may occur with implant construction. *(d) What might go wrong with flaps?* Description of complications that may arise from flap reconstruction, such as muscle weakness, abdominal hernias, wound-healing problems, seroma, etc. *(e) Other risks—What happens if you are a smoker?* Description that women are more likely to experience complications after flap reconstruction. *(f) Other risks—What happens if you are obese?* Description that women who are obese often experience more complications with flap surgery.
**Section 4** **Other questions**	*(a) What will the reconstructed breast feel like?* Description of the outcomes of having breast reconstruction including the reconstructed breast not having the same feeling or sensation as before, not being able to breast feed, scarring, and the possibility of discomfort for a few months post-surgery. *(b) What will the reconstructed breast look like?* Description of how the reconstructed breast will look including a noticeable difference when undressed, the breast reconstruction enabling most types of clothing to be worn including swimwear, and differences in appearance after flap reconstruction compared with an implant. *(c) Reconstruction results* Lists the factors that will determine reconstruction results such as health, type of mastectomy/reconstruction, type/size of implant, chest structure and body shape, shape of other breast, amount of tissue available for flap reconstruction, body’s ability to heal, and skill/experience of surgical team. *(d) How long will I take to recover?* Mentions the typical recovery period post-surgery (2–3 weeks after implant reconstruction, up to 6 weeks for flap procedure), the doctor may provide pain medication, possible hospital stay between 2 and 7 days, and the use of surgical drains to remove excess fluid.
**Section 5** **What should I know before making a decision?**	*(a) Weighing up the choices* Brief opening statement about the recommendation to carefully weigh up the advantages and disadvantages of each option. **Weighing reconstruction versus no reconstruction** *(b) Advantages of breast reconstruction* List of the advantages of undergoing breast reconstruction, such as restoring breast shape/contour, being able to wear most types of clothing including swimwear, feeling better about the way they look, etc. *(c) Advantages of no breast reconstruction* List of the advantages of not having breast reconstruction, such as not needing additional breast surgery, experiencing fewer post-surgery complications, and faster recovery time. *(d) Disadvantages of breast reconstruction* List of the disadvantages of breast reconstruction, such as a change in the appearance and shape of the breast, longer surgery and recovery times, etc. *(e) Disadvantages of no breast reconstruction* List of the disadvantages of no breast reconstruction, such as feeling unbalanced, needing to wear a breast prosthesis, restrictions on clothing, etc. **Weighing implant versus flap reconstruction** *(f) Implant reconstruction* List of the advantages and disadvantages associated with implant reconstruction. *(g) Flap reconstruction* List of the advantages and disadvantages of flap reconstruction. **Weighing immediate versus delayed reconstruction** *(h) Immediate reconstruction* List of the advantages and disadvantages of immediate reconstruction. *(i) Delayed reconstruction* List of the advantages and disadvantages of delayed reconstruction. **Exercises** Comprises some exercises relating to the considerations of the above advantages and disadvantages. The exercises include (1) “Consider the reasons for choosing between an implant or a flap type/immediate or delayed surgery. Please tick the relevant boxes indicating how important each aspect is to you”, and (2) “My current thoughts about breast reconstruction: Leaning towards having breast plant reconstruction, Undecided, Leaning towards flap breast reconstruction”.

## Appendix B. Think-Aloud Interview Schedule (Phase 1: Vietnamese-Speaking Women)

### Appendix B.1. Practice Task

To help you get used to thinking out loud, we will do a practice exercise first, which is not related to breast reconstruction. Then, we will go to the booklet for breast reconstruction.

Practice Task

*** START RECORDER***

Now we will do the practice task, which is a short cooking recipe.

Start when you are ready and remember to read the recipe out loud and say what your thoughts are as you go through it and at the end of the recipe.

[Prompt to keep talking and give feedback on thinking-aloud frequency at the end.]

### Appendix B.2. Cognitive Interview

Now let us move to the Vietnamese booklet.

**Section 1:**

*Please start reading the booklet from the cover page through to the end of Section 1. Feel free to say your thoughts aloud as you are reading the section, for example, if you’re thinking something is particularly interesting, or you’re not too sure what something means as you are reading it, then say that aloud. After you finish reading this section, just say anything that pops into your head so we can get some understanding of what you thought about both the information in this section and also how it was presented*.

[Prompt the participant to keep talking and clarify the information she is providing when necessary]

Questions after Section 1:

- What do you think about the cover?
- What is Section 1 telling you?
- How interesting did you find the information in this section? Which parts of the section were the most interesting to you?
- Were you confused by anything in this section (information and/or images)? If yes, what in particular?
- What is your understanding of the images in Section 1?
- What do you think about the layout of the section? What did you like and not like?
- Is there anything else that you think should be included about this particular topic?

**Section 2:**

*Now, let us move to the next section and repeat the same thing we just did. Please have a read through this section and then tell me about your thoughts*.

[Prompt the participant to keep talking and clarify the information she is providing when necessary]

Questions after Section 2:

- What is Section 2 telling you?
- How interesting did you find the information in this section? Which parts of the section were the most interesting to you?
- Were you confused by anything in this section (information and/or images)? If yes, what in particular?
- What is your understanding of the images in Section 2?
- What do you think about the layout of the section? What did you like and not like?
- Is there anything else that you think should be included about this particular topic?

**Section 3:**

*Now, let us move to Section 3.*

[Prompt the participant to keep talking and clarify the information she is providing when necessary]

Questions after Section 3:

- What is Section 3 telling you?
- How interesting did you find the information in this section? Which parts of the section were the most interesting to you?
- Were you confused by anything in this section (information and/or images)? If yes, what in particular?
- What is your understanding of the images in Section 3?
- What do you think about the layout of the section? What did you like and not like?
- Is there anything else that you think should be included about this particular topic?

**Section 4:**

*Now, let us move to Section 4.*

[Prompt the participant to keep talking and clarify the information she is providing when necessary]

Questions after Section 4:

- What is Section 4 telling you?
- How interesting did you find the information in this section? Which parts of the section were the most interesting to you?
- Were you confused by anything in this section (information and/or images)? If yes, what in particular?
- What is your understanding of the images in Section 4?
- What do you think about the layout of the section? What did you like and not like?
- Is there anything else that you think should be included about this particular topic?

**Section 5:**

*Finally, let us move to the fifth section. Can you please confirm that you have done this part prior to this interview?*

- *Yes → Great! Let us move to the questions then.*
- *No → OK, how about I hang up now and give you 15 min to do this part? I will call you back after about 15 min to continue with the questions.*

Questions after Section 5:

- What are your thoughts about the decision-making tasks in this section?
- What are your thoughts about the quotes from other women who were also deciding about breast reconstruction?
- Did this section help you decide about whether breast reconstruction is right for you?

## Appendix C. Semi-Structured Interview Guide (Phase 2: Arabic-Speaking Women)

### Appendix C.1. Demographic Information

(a) How old are you?
(b) When were you diagnosed with breast cancer?
(c) (If diagnosed with breast cancer previously) How old were you when you had your mastectomy?
(d) What is your current relationship status? (married, single, divorced or separated, widowed)
(e) What country were you born in?
(f) If not born in Australia, what year did you first come to live in Australia?

### Appendix C.2. Arabic Breast Reconstruction Decision Aid

*Overall impression*

1. What did you think of this booklet? [Prompt: what did you like or dislike about it? what part did you find easy or difficult to understand?]

*Understanding of content*

1. What does breast reconstruction mean to you?
2. What is the difference between implant and flap reconstruction? What is the difference between immediate and delayed reconstruction?

*Usability*

1. Which parts of the booklet did you find easiest to understand?
2. Which parts were most confusing or difficult? For each part that was confusing, what did you find to be confusing and how can we make it clearer?
3. What did you think of the way that the information was presented? What would you recommend changing?
4. Is there any other information that you think should have been included?
5. How useful is this decision aid for helping women understand and decide about breast reconstruction?
6. When do you think it should be given to patients? [Prompts: How would you prefer to access the decision aid? Explore options of hospital waiting rooms, clinicians, community centres, online]

*Format of booklet*

7. What did you think of the overall length of the booklet? [Prompts to explore response: sections which should be deleted/shortened? sections that need more information?]
8. What did you think of the cover page and headings?
9. What did you think of the illustrations? Would you prefer real photographs? [Prompts: what do you like about the images? what don’t you like?]

*Language*

10. What did you think of the translation? Were there grammatical issues?
11. How did you go with understanding the medical terminology? [Prompt: what would you recommend changing?]
12. How suitable do you think this decision aid is for people from your cultural background or community?

**Easy Arabic booklet**

1. What did you think of this Easy Arabic booklet? What did you like about it? What didn’t you like about it?
2. Is there further information you think that should be added?
3. How useful is it in comparison with the more detailed decision aid? [Prompt: in what way is this more useful/less useful?]

### Appendix C.3. Preparation for Decision-Making Scale

I’m going to ask you some questions now about your thoughts on the effect of the booklets.

For each question, I’ll ask you to rate your opinion on a scale from 1 to 5. The ratings are

1—Not at all
2—A little
3—Somewhat
4—Quite a bit
5—A great deal

Did this educational material:

1. Help you recognise that a decision needs to be made?
2. Prepare you to make a better decision?
3. Help you think about the pros and cons of each option?
4. Help you think about which pros and cons are most important?
5. Help you know that the decision depends on what matters most to you?
6. Help you organise your own thoughts about the decision?
7. Help you think about how involved you want to be in this decision?
8. Help you identify questions you want to ask about your doctor?
9. Prepare you to talk to your doctor about what matters most to you?
10. Prepare you for a follow-up visit with your doctor?
