# Supplementary material for: To Reconstruct or Not to Reconstruct: Piloting a Vietnamese and Arabic Breast Reconstruction Decision Aid in Australia
Source: Curr Oncol. 2024 Jun 28;31(7):3713–37. doi: 10.3390/curroncol31070274 (PMC11275298; doi:10.3390/curroncol31070274)
Supplement: Supplementary file 1 [file curroncol-31-00274-s001.zip › CALD_DA_Supplementary File 1.pdf]

Supplementary Table S1. Additional Themes identified in Phase 2 (Arabic-speaking women)

Themes, sub-themes, and description
**1. Perceptions of breast cancer (BC) and breast reconstruction (BR)**<br>**1.1. Coping strategies/mechanisms employed by target group in response to cancer**<br>Core coping mechanisms that our target group used in response to cancer and through their decision-making process include adjusting to the situation, using various sources of support such as their family member or in-language doctors, seeking psychological support, seeking social support through BC support groups, and using spirituality as a source of strength.<br>*"When the person who hears about the misfortune/ adversity of others… his misfortune/adversity becomes more tolerable" (A009).*<br><br>**1.2. End of the world**<br>One participant highlighted that when women who have limited knowledge of BC is first diagnosed, they may feel like this means the end of the world. Having access to BR related material earlier on, can help women better understand what they are experiencing.<br>*The end of the world… depending on what situation I went through… for example… when they first told me that you have cancer… I said the most I will last is 6 months or 1 year… (A009)*<br><br>**1.3. Negative impact on mental health**<br>BC can result in a negative impact on mental health. As a result, taking this into account and including a section on mental health/being sensitive to how information is presented was regarded as important.<br>*Because there is a little of depression… (A010)*<br><br>**1.4. Unexpected surprises**<br>One participant indicated that accessing BC and BR materials in the Arabic language can help a woman understand the issue in more detail and not experience unexpected surprises e.g. timing for recovery in treatment phases.<br>*I was surprised by its look…surprised... that is… when I took it… it helped me a little when I read, and I understood… that is… what is the reason…. That is…. why this is happening to me…. (A008)*<br><br>**1.5. Varying meanings associated with BR**<br>The participants touched upon various meanings associated with BR which was further enhanced by them reading the Arabic DA and easy English/Arabic Info sheet. Some felt that BR was more related to being a cosmetic surgery. Other indicated that BR provided better spirits for women, more positive body image associations, impacted positively on a woman's psychosocial wellbeing, restored a sense of femininity, and offered a chance for rebuilding and renewal of the impacted area.<br>*It is a nice thing that a woman … that is losing a section… that is us woman…. You know we take pride in the breast and that…… (A009)*
**2. Facilitators and barriers to breast reconstruction (BR) decisions**<br>**2.1. Facilitators for BR**<br>One participant commented that they were more likely to undertake BR if they are not going to take from other body parts, rather from the breast area itself. Another participant

Themes, sub-themes, and description
noted how interviews such as the one we conducted can help in facilitating and encouraging women to undertake BR.<br>*With the interview… they are encouraging…the patient to do the surgery. (A009)*<br><br>**2.2. Barriers to undergoing BR**<br>Several participants discussed barriers to be undertaking BR. Some of the barriers we identified from the analysis include: a need to undertake an additional surgery, concerns related to the quality of the treatment, experiencing surprises not predicted earlier on, lengthy recovery time, prioritisation of family, and still undergoing chemotherapy. Interestingly, participant stage of life also impacted choice related to BR. Some of the participants that were in the older age group felt that if they were younger, they would have had a bigger desire to undertake BR, especially when they considered the risks associated with further surgery.<br>*So, I said, I do not want to handle this risk…. that the surgeon told me about… Because I mean…. I handled a lot in my life. I said I do not want to handle anymore… What is left in life is not more than what passed already (Arabic saying) (A003)*
**3. Decision-making approaches within the community**<br>**3.1. Confident in their decision to not undergo reconstruction**<br>Some of the participants noted that they were confident in their decision not to undertake BR. After reading the DA and assessing their own situation, they felt that the DA confirmed that their choice to not undertaken BR was the better option for them.<br>*From my experience, me and what I have been through…… it did not help me, because I still remain with my previous decision. (A003)*<br><br>**3.2. Decision-making based on personal preferences and circumstances**<br>Another crucial factor related to BR decision-making is the importance of personal preferences of patients. One of the respondents highlighted that the choice to undertake BR is based on what each woman prefers. There is no one choice fits all. Some women may feel that they want to return the section in the body that was removed, whilst others that are going through different circumstances (e.g. being older in age or not wanting to undertake further surgery) may feel that they do not want BR. Similarly, the choice between immediate and delayed BR is dependent on the circumstance of each woman.<br>*Let the person finish from the surgeries and everything and the radiation and then… they can do BR (A009)*<br><br>**3.3. Familial process/Seeking opinions of multiple people**<br>One of the respondents noted the importance of decision-making being based on a familial process. Family support networks can contribute to reassurance in decision making related to BR and help in evaluating if BR is something that a patient is considering.<br>*So, I gave it to people that are important to me, so that they can also read and see it for me. What is good for me and what is not. (A002)*<br>Moreover, the participants stressed the importance of seeking multiple opinions, to ensure that a patient who is taking part in this difficult decision does not feel isolated.<br>*"What I mean, is two opinions are better than one opinion". (A002)*<br><br>**3.4. Impact of healthcare professionals' support on target group/reliance on doctor's expertise**<br>Several participants touched upon the importance of health professionals supporting the patient who is considering BR or other BC related treatments. Having the correct contact

Themes, sub-themes, and description
person in the decision-making process is complementary to receiving the decision aid booklets.<br>*This is in my opinion. Ahh… because I asked my doctor that day… She said she can't do it for me…. Guess why? Because I wanted to do … Ahh… radiotherapy…. And she said that the radiotherapy… If I do it after the surgery, it affects the implants…. (A003)*<br>Some clients really valued expert doctor advise. One of the participants gave her opinion about treatment options prior to BR and later said that the doctor has more knowledge, so she prefers to rely on their advice when making a final decision.<br>*But they… the doctors… of course they know more (A009)*<br><br>**3.5. Preference for patient-centered decision making**<br>Some participants indicated that they would have preferred that patient options are kept open, and the surgeon really taps into what the end goal of the client is. This means that all possible options of BR should be discussed with the patient prior to undertaking mastectomy, and this can be better managed through accessing the DA and following up with the surgeon regarding queries. This impacts acceptability of the DA more favourably.<br>*I said…. …. Later… But I don't think that she took into account… Ahh… In that she kept more skin or things like that…. (A006)*
