# Supplementary material for: To Reconstruct or Not to Reconstruct: Piloting a Vietnamese and Arabic Breast Reconstruction Decision Aid in Australia
Source: Curr Oncol. 2024 Jun 28;31(7):3713–37. doi: 10.3390/curroncol31070274 (PMC11275298; doi:10.3390/curroncol31070274)
Supplement: Supplementary file 1 [file curroncol-31-00274-s001.zip › CALD_DA_Supplementary File 2.pdf]

Figure S1. Examples of decision aid content (English)

Things to think about	Very important to me	Slightly important to me	Not important to me
If I **have breast reconstruction**, I will have a breast shape without having to wear an artificial breast form.<br><br>To me, having a breast shape without having to wear an artificial breast form is:			
If I **have breast reconstruction**, I will be able to wear most types of clothing.<br><br>To me, being able to wear most types of clothing is:			
If I **don't have breast reconstruction**, I won't need more breast operations.<br><br>To me, not having more breast operations is:			

My current thoughts about having breast reconstruction: I am.....

|________________________________|________________________________|

Leaning towards having breast reconstruction | Undecided | Leaning towards NOT having breast reconstruction

Figure S2. Examples of decision aid content (Vietnamese)

Những việc cần xem xét	Rất quan trọng đối với tôi	Hơi quan trọng đối với tôi	Chẳng quan trọng gì cả đối với tôi
Tái tạo bằng cách độn ngực nhân tạo thì ít phức tạp về mặt phẫu thuật so với tái tạo vạt. Tôi thường sẽ hồi phục nhanh sau cuộc giải phẫu độn ngực nhân tạo. Tôi cảm thấy rằng việc chọn phẫu thuật tái tạo mà giúp tôi hồi phục càng nhanh càng tốt là:			
Nếu tôi chọn cách tái tạo vú bằng túi độn nhân tạo, tôi sẽ không cần dùng đến các mô tế bào tự thân để tái tạo vú mới. Tôi cho rằng việc không cần dùng đến phần nào khác của cơ thể mình để tái tạo vú là điều:			
Nếu chọn cách tái tạo vú bằng cách dùng vạt da/cơ, thì vú tái tạo của tôi có thể có hình dáng/cảm giác tự nhiên hơn là dùng túi độn nhân tạo. Tôi cho rằng việc vú tái tạo có vẻ ngoài/cảm giác tự nhiên là điều:			
Nếu chọn cách tái tạo vú bằng cách dùng vạt da/cơ, thì kích cỡ vú tái tạo của tôi sẽ thay đổi khi tôi tăng cân hoặc giảm cân. Tôi cho rằng việc kích cỡ vú thay đổi khi tôi tăng cân/giảm cân là điều:			
Giải phẫu tái tạo bằng vạt da/cơ có nghĩa là tôi sẽ có thêm các vết sẹo ở nơi mô tế bào bị lấy đi. Tôi cho rằng cách chọn để ít bị các vết sẹo thêm nữa trên cơ thể của tôi là điều:			
Nếu tôi có phẫu thuật tái tạo ghép vạt trì hoãn, thì thường màu da của vú tái tạo sẽ khó giống màu da của vú còn lại. Với phẫu thuật túi độn nhân tạo thì màu của cả hai vú sẽ giống nhau. Tôi cảm thấy rằng việc hai vú có màu da giống nhau là điều:			
Lý do khác:			
Lý do khác:			

Suy nghĩ hiện thời của tôi về việc tái tạo vú: Tôi đang.....

Có khuynh hướng thích tái tạo vú bằng cách dùng túi độn nhân tạo	Chưa quyết định	Có khuynh hướng thích tái tạo vú bằng cách dùng vạt

Figure S3. Examples of decision aid content (Arabic)

الأشياء التي يجب التفكير فيها	مُهمّاً جدا بالنسبة لي	أقل أهميّة بالنسبة لي	ليس مهماً بالنسبة لي
إذا اخترتُ إعادة البناء الفوريّة، يتم هذا في نفس وقت استئصال الثدي، وسوف أستيقظ بعد الجراحة ببعض الشكل للثدي.<br><br>بالنسبة لي، الحصول على شكل جديد للثدي بأسرع ما يمكن هو:			
إذا اخترتُ إعادة البناء الفوريّة، قد يكون من المُمكن الحفاظ على جلد ثديي وحلمتي.<br><br>بالنسبة لي، الحفاظ على جلد الثدي والحلمة هو:			
إذا اخترتُ إعادة البناء الفوريّة، قد تكون تكاليف الجراحة أقل حيث سيتم إجراء استئصال الثدي وإعادة البناء في نفس الوقت.<br><br>بالنسبة لي، فإن تقليل تكاليف الجراحة هو:			
إذا اخترتُ إعادة البناء المؤجّلة، سأحظى بوقت أسرع للشفاء من جراحة استئصال الثدي فقط.<br><br>بالنسبة لي، الحصول على وقت شفاء أسرع من الجراحة هو:			
إذا اخترتُ إعادة البناء المؤجّلة، سيكون لديّ المزيد من الوقت لإتخاذ قرار.<br><br>بالنسبة لي، فإن وجود وقت أطول ومعقول لاتخاذ قرار بشأن إعادة البناء هو:			
إذا اخترتُ إعادة البناء المؤجّلة، سيكون هناك خطرٌ أقل لحدوث مُضاعفات جراحية قد تتسبّب في تأخير أي علاج آخر مثل العلاج الكيميائي.<br><br>بالنسبة لي، فإن القُدرة على بدء أي علاج آخر في أسرع وقت مُمكن هي:			
سبب آخر:			
سبب آخر:			

أفكاري الحالية حول توقيت إعادة بناء الثدي:

أميل نحو إعادة بناء الثدي الفوريّة — غير مُحَدّدَة الرأي — أميل نحو إعادة بناء الثدي المؤجّلة

Figure S4. Easy English/Arabic infographic content

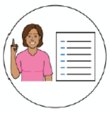

Hard words

This guide has some hard words.

The first time we write a hard word

- the word is in **blue**
- we write what the hard word means.

You can get help with this guide

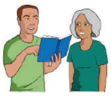

You can get someone to help you

- read this guide
- know what this guide is about

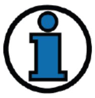

- find more information.

اذا كان لديك صعوبات بالنطق أو السمع

استخدمي خدمات National Relay Service.

اتصلي على 1800 555 660
زودي الموظف برقم الهاتف الذي تريدين الاتصال به.

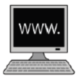

communications.gov.au/accesshub/nrs

تم تمويل دليل إعادة بناء الثدي بفخر من قِبل معهد السرطان NSW. هذا الدليل هو للإستعمال الشخصي فقط، و ليس للأغراض التجارية. تم إعداد اللغة الإنجليزية المبسطة في هذا الإصدار عن طريق سكوب (استراليا) المحدودة (scopeaust.org.au) وفقاً للمحتويات التي تم تزويدها من خلال مقاطعة جنوب غربي سيدني المحلية للصحة ومعهد إنجهام Ingham Institute، تموز/يوليو 2021. و تم ترجمتها بعد ذلك للغة العربية. الرسوم عن طريق جولي هايسوم. Picture Communication Symbols ©1981–2021 لتوبي دايناقوكس. جميع الحقوق محفوظة حول العالم. تم استعمال الدليل بعد أخذ التفويض بذلك. Boardmaker® بوردميكر هي علامة تجارية تابعة لتوبي دايناڤوكس. لا يجوز بيع هذا الدليل الى طرف ثالث. لا يجوز إعادة إستعمال هذه الصور بدون إذن.

Breast reconstruction after your mastectomy

After your mastectomy you can have a breast reconstruction with

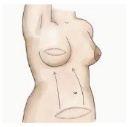

- your body tissue
  - for example, muscle, fat and skin

or

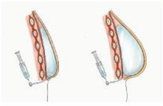

- a breast implant called a **tissue expander**.

A tissue expander is a pocket that can be filled with salt water.

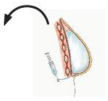

When the tissue expander has stretched your skin you will have another operation to

- take the tissue expander out

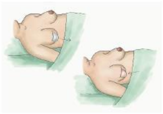

- put a new breast implant in.

عملية إعادة بناء الثدي بعد عملية الأستئصال

بعد الخضوع لعملية استئصال الثدي يمكنك إعادة بناء للثدي باستخدام

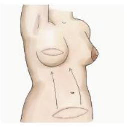

- أنسجة جسمك
  - على سبيل المثال، العضلات، والدهون، والجلد

أو

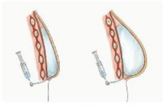

- تسمى زراعة الثدي **موسع أنسجة**.

موسع الأنسجة هو عبارة عن كيس يتم حشوه بماء مملح.

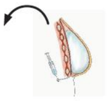

عندما يقوم موسع الأنسجة بتمديد جلدك، ستخضعين لعملية أخرى

- لإزالة موسع الأنسجة

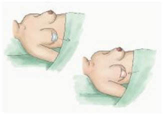

- و وضع حشوة ثدى جديدة.
